# Parliament and the Profession

**Published:** 1917-03-17

**Authors:** 


### Parliament and the Profession.

### Supply of Nurses.

Mr. Macpherson informed Mr. MacVeagh that the report of the Committee on the supply of Nurses had been received by the War Office, and after it had been considered by the Army Council will be laid upon the table.

### State Registration of Nurses.

Mr. Hayes Fisher, Parliamentary Secretary to the Local Government Board, informed Mr. MacCallum Scott that full consideration will be given to any representations made by organised societies of nurses before the Government introduces any Bill for the State Registration of Nurses. The introduction of such a Bill is still undergoing consideration.

### Military Service Hospital Employees.

Mr. Macpherson informed Mr. Hohler that it was expected that men fit for general service employed at No. 2 General Hospital, London, and other military hospital as dispensers, linen-storekeepers, and orderly-room clerks would shortly be released.

### The Great Northern Central Hospital and Spirit Supply.

Sir G. Touche, in a question to the Secretary to the Treasury, drew attention to the difficulty experienced by the Great Northern Central Hospital, where the consumption of brandy is two gallons every ten days, in obtaining supplies owing to the nature of the restrictions governing the withdrawal of spirits from bond. Mr. Baldwin said that the Board of Customs and Excise had had no representations made to them that the present restrictions were inconveniently affecting hospitals, and he promised to inquire further into the matter.

